# Optimal Cooperative Guidance Laws for Two UAVs Under Sensor Information Deficiency Constraints

**DOI:** 10.3390/s20174790

**Published:** 2020-08-25

**Authors:** Daniel Lee, Han-Lim Choi, Jong-Han Kim

**Affiliations:** 1 Department of Aerospace Engineering, Korea Advanced Institute of Science and Technology, Daejeon 34141, Korea; dilee@lics.kaist.ac.kr (D.L.); hanlimc@kaist.ac.kr (H.-L.C.); 2 Department of Electronic Engineering, Kyung Hee University, Yongin 17104, Korea

**Keywords:** UAVs, cooperative guidance, networked systems control, decentralized optimal control

## Abstract

This paper presents closed-form optimal cooperative guidance laws for two UAVs under information constraints that achieve the required relative approach angle. Two UAVs cooperate to optimize a common cost function under a coupled constraint on terminal velocity vectors and the information constraint which defines the sensor information availability. To handle the information constraint, a general two-player partially nested decentralized optimal control problem is considered in the continuous finite-horizon time domain. It is shown that under the state-separation principle the optimal solution of the decentralized control problem can be obtained by solving two centralized subproblems which cover the prediction problem for the information-deficient player and the prediction error minimization problem for the player with full information. Based on the solution of the decentralized optimal control problem, the explicit closed-form cooperative guidance laws that can be efficiently implemented on conventional guidance computers are derived. The performance of the proposed guidance laws is investigated on both centralized and decentralized cooperative scenarios with nonlinear engagement kinematics of networked two-UAV systems.

## 1. Introduction

### 1.1. Cooperative Control of Networked Systems

Cooperative control problems in networks of multiple autonomous agents have received considerable attention in civilian and military applications. This is due to the advantages that swarm of multi-agent system brings and the growing interest in understanding the tactical hunting behaviors of animal group that realize greater efficiency and operational capability. Especially for cooperative missions of multiple unmanned aerial vehicles (UAVs), cooperative control techniques can be used to improve the operational performance and survivability, as well as greatly reducing the overall effort that would have been previously required by independently operating multiple agents for attack or surveillance missions. For instances, cooperative attack techniques are devised as a countermeasure against the formidable defense systems [1,2,3] and cooperative surveillance techniques are adopted in various applications in order to broadening the time and space coverage of monitoring and detection [4,5,6].

To achieve high-level autonomy for cooperative UAVs, one of the fundamental capabilities is to approach the destination with relative geometric constraints (e.g., terminal time and angle). For instance, the terminal time and angle constraints are the fundamental components that lead the cooperative loitering munitions to saturate and penetrate the defense systems [2]. Also they perform the key role in cooperative surveillance missions by enhancing the observability of the multi-measuring environment [7] and maximizing the mutual information obtained by multiple sensors [8,9]. Even though there have been lots of studies on this domain, complicated numerical trajectory optimization techniques are usually used to obtain the optimal time or energy path with the geometric constraints [10,11,12]. Considering the computational capacity of conventional guidance computers, numerical optimization techniques might not be suitable to swarm UAV systems as the computational complexity typically increases with the number of UAVs. Therefore, adopting analytical guidance algorithm can lead more efficiency for cooperation of multiple UAVs [13].

### 1.2. Related Works

The most widely accepted approach for deriving analytical guidance algorithms is based on the linear optimal control theory, e.g., linear quadratic regulator (LQR), which gives the optimal control input in a state-feedback form [14,15]. There have been many studies on cooperative guidance problems. To control the terminal time of multiple vehicles [1,3] have proposed impact-time control guidance (ITCG) and cooperative proportional navigation (CPN) which synchronize the time-to-go of each vehicle. The ITCG controls the impact time of vehicles to a predetermined desired value and CPN decreases the variance of time-to-go during the homing. To control the impact angle of multiple vehicles, there have been lots of studies and application such as the impact-angle control guidance (IACG) techniques [16,17,18]. The IACG independently guides each of vehicle to a predetermined desired value based on the one-on-one solution. Furthermore, studies have been extended to control the impact time and angle simultaneously (ITACG) [2,19,20].

The aforementioned cooperative guidance laws can be categorized by two types, which are the implicit and explicit cooperation. In implicit cooperation, multiple vehicles are guided to the target without an information sharing networks but with predetermined objectives (e.g., ITCG, IACG), while explicit cooperation shares information to optimize a common team criteria (e.g., CPN). Compared to the impact-time control guidance laws, for the impact-angle control guidance laws it is not common to construct a closed-loop structure using communication networks. Shaferman and Shima [21] proposed an explicit cooperative guidance law for controlling the relative impact angle of multiple vehicles for any team size which provides substantially better results than implicit guidance laws in the acceleration requirements aspect.

However, networked systems should be considered from a realistic point of view, such as failure or security issues that could seriously impact the system. The connectivity of networked systems becomes a challenge for long-distance applications [4] and they are prone to malicious attacks as the network size increases [22]. To overcome such challenges, [23] has proposed the emergent self-organization algorithm where the strategies of the cooperative UAVs depends only on locally available information while providing robust and dependable connections on UAV-relay networks and [24] has proposed a distributed matching game model where the source UAVs select the preferred relay UAVs competitively, according to their own transmission requirements. In [25] a flight planning procedure is addressed for maintaining the connectivity in multi-UAV swam sensing missions where the message passing procedure on decentralized coordination algorithm is used for propagating information in an on-line learning approach. For maintaining and tracking the network connectivity throughout the formation process of multiple UAVs, [26] presents a decentralized controller which provides a target-centric formation.

The key approach of the aforementioned studies is handling the network with limited information which can be described in information constraints, in optimal control problems. Accordingly, the control problems with network under information constraints should be considered. Such problems can be distinguished as either centralized or decentralized depend on the network structure. In centralized problems, systems involve a single decision maker. This may be because there is only one system involved or multiple subsystems might communicate to the central processor which decides the decisions for the overall system. By contrast, decentralized problems are basically defined as any system which is not centralized. Intuitively, one might think of a system in which each subsystems have their own processing unit and make their own decisions based on their own measurements [27]. Although the optimal solution of centralized control problems are well known, it is hard to obtain the optimal solution of decentralized problems. The well-known example of Witsenhausen [28] showed that the optimal controller of decentralized system with feedback is generally nonlinear and computationally intractable. Ho and Chu [29] developed a class of information structure for decentralized control problems, called partially nested, for which the optimal controller is linear. A broader class of problems called quadratically invariant was developed by Rotkowitz et al. [30] which includes the partially nested systems and have the property that the set of closed-loop maps is convex [31]. The explicit optimal solution of quadratically invariant problems are obtained in various search space. Swigart et al. [32] attained an explicit state-space solution in the discrete finite-horizon time domain, using a spectral factorization approach and dynamic programming. Kim and Lall [33,34,35,36] attained the explicit solution in the continuous infinite-horizon time domain by defining a unifying condition that split the decentralized optimal control problem into multiple centralized problems. However, to apply the decentralized solution to the cooperative guidance laws given as a polynomial function of time-to-go, it is necessary to obtain an explicit solution in the continuous finite-horizon time domain.

### 1.3. Contributions of This Paper

This paper aims to obtain the explicit guidance laws to control the relative approach angle of two UAVs under the nested dynamical structures with sensor information constraints. The relative approach angle constraints and the information constraints are considered to enhance the observability of networked two-UAV systems on the target and to cover the failure or security issues on networked systems, respectively. The centralized and decentralized optimal guidance solutions of networked two-UAV systems are derived in the continuous finite-horizon time domain. The key difference from the conventional LQR-based optimal guidance laws is that our approach considers the information deficiency constraints between the two UAVs, i.e., the first UAV’s sensor measurement is available to the second UAV (via communication networks or by direct measurements), so the second UAV can use the first UAV’s information for cooperation, while the first UAV is not able to use the second UAV’s measurements. Please note that the conventional LQR frameworks are not able to handle these information constraints. Motivated by [35], the state-separation principle is proposed which enables separation of the decentralized control problem into multiple centralized problems. Based on the optimal solution of the decentralized control problem, the explicit closed-form cooperative guidance solutions are derived in terms of the line-of-sight angles and the line-of-sight angle rates, thus it can be easily implemented on typical guidance computers. To the best knowledge of the authors, this is the first attempt to describe the closed-form cooperative guidance solution that explicitly minimizes the finite-horizon linear quadratic objective function under information constraints. Finally, the solutions are converted to the guidance form with the cost function with the terminal velocity constraint.

The remainder of this paper is organized as follows: the two-UAV cooperative engagement geometry is presented first, and the optimal control problem under the nested dynamical structures with information constraints are formulated in Section 2. Section 3 shows the solution and the proofs of the decentralized control problem followed by the derivation of the cooperative guidance laws. In Section 4, numerical simulation results of networked two-UAV systems are presented. The concluding remarks are given in Section 5.

## 2. Problem Statement

**Figure 1:**
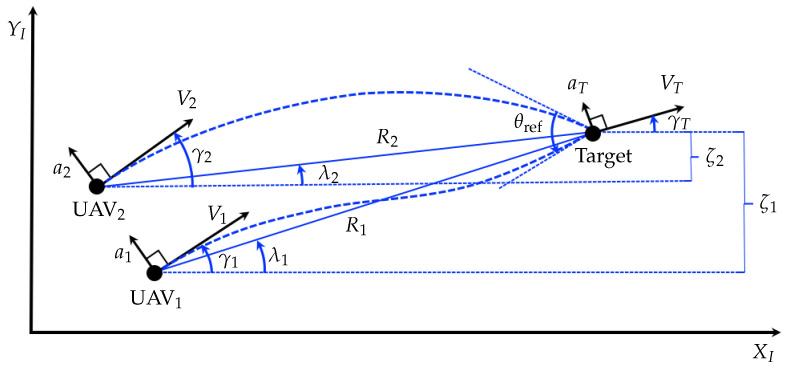
Two UAVs in cooperative engagement geometry.

Let us consider the planar homing guidance geometry of two UAVs and a stationary (or slowly moving) target, as shown in Figure 1. The $X_{I}-Y_{I}$ frame is an inertial Cartesian coordinate system which is fixed in space. Variables associated with the *i*-th UAV for i=1,2 and the target are denoted by subscripts *i* and *T*. Here V,γ and λ denote the velocity, flight-path angle and the line-of-sight (LOS) angle, respectively. The normal acceleration of each vehicle is denoted by *a* and the predetermined relative approach angle constraint of two UAVs are denoted by $\theta_{\mathrm{ref}}$. The relative distance of each UAV and target in $Y_{I}$ axis is denoted by $\zeta_{i}$. Other variables in Figure 1 are self-explanatory.

### 2.1. Kinematics Relations for Cooperative Engagement

The kinematics of each UAV for the homing problem can be expressed in vector form as follows:

(1) $$\begin{array}{c} \begin{array}{cc} R_{i} & =R_{i}\cos\lambda_{i}{\hat{ı}}_{x}+R_{i}\sin\lambda_{i}{\hat{ı}}_{y}\\ {\dot{R}}_{i} & =\left(-V_{i}\cos\gamma_{i}+V_{T}\cos\gamma_{T}\right){\hat{ı}}_{x}+\left(-V_{i}\sin\gamma_{i}+V_{T}\sin\gamma_{T}\right){\hat{ı}}_{y}\\ a_{i} & =\left(-a_{i}\sin\gamma_{i}-a_{T}\sin\gamma_{T}\right){\hat{ı}}_{x}+\left(a_{i}\cos\gamma_{i}+a_{T}\cos\gamma_{T}\right){\hat{ı}}_{y} \end{array} \end{array}$$

where the variables in bold font represent the value in $X_{I}-Y_{I}$ coordinate. Here ${\hat{ı}}_{x}$ and ${\hat{ı}}_{y}$ are the unit vectors of $X_{I}$ axis and $Y_{I}$ axis and the velocities of each UAV and target are assumed to be constant. Under the assumption, the constant closing velocity $V_{c}$ and the interception time can be calculated as

(2) $$\begin{array}{cc} V_{c,i} & =-∥{\dot{R}}_{i}∥\\ & =V_{i}\cos\left(\gamma_{i}-\lambda_{i}\right)-V_{T}\cos\left(\gamma_{T}-\lambda_{i}\right)\\ t_{f,i} & =t_{0}+\frac{R_{i}\left(t_{0}\right)}{V_{c,i}} \end{array}$$

where $R_{i}\left(t\right)$ is the nominal range-to-go of the *i*-th UAV at time *t*, and $t_{0}$ is the initial time.

The homing kinematics in Equation (1) is clearly nonlinear, which needs to be linearized in order for deriving guidance laws based on the linear quadratic (LQ) optimal control theory. For this purpose, the near-collision course assumption, which is valid for small $\gamma_{t}$ and $\lambda_{i}$, is used. Then the linearized kinematics of two UAVs can be expressed in state-space form as follows:

(3) $$\dot{x}=Ax+Bu+w\;$$

where

$$\begin{array}{c} A=\left[\begin{array}{cccc} 0 & 1 & 0 & 0\\ 0 & 0 & 0 & 0\\ 0 & 0 & 0 & 1\\ 0 & 0 & 0 & 0 \end{array}\right],\;B=\left[\begin{array}{cc} 0 & 0\\ 1 & 0\\ 0 & 0\\ 0 & 1 \end{array}\right],\;x≜\left[\begin{array}{c} \zeta_{1}\\ {\dot{\zeta }}_{1}\\ \zeta_{2}\\ {\dot{\zeta }}_{2} \end{array}\right],\;u≜\left[\begin{array}{c} a_{1}\\ a_{2} \end{array}\right] \end{array}$$

with the process noise *w* which covers the wind gust or the target maneuver $a_{t}$. Please note that this is a straight-forward two-agent extension of the very widely used linearization techniques for classical optimal guidance problems [37,38,39].

### 2.2. Nested Dynamical Systems with Sensor Information Deficiency Constraints

**Figure 2:**
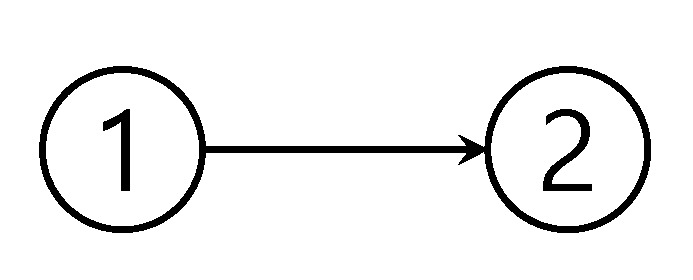
Two-player nested dynamical structures.

Let us consider the nested dynamical structures for two UAVs, as shown in Figure 2 which implies that the dynamical interactions are directional. Without loss of generality, we consider two interconnected linear systems with nested dynamics as follows:

(4) $$\begin{array}{cc} \; & \left[\begin{array}{c} {\dot{x}}_{1}\\ {\dot{x}}_{2} \end{array}\right]=\underset{A}{\underbrace{\left[\begin{array}{cc} A_{11} & 0\\ A_{21} & A_{22} \end{array}\right]}}\left[\begin{array}{c} x_{1}\\ x_{2} \end{array}\right]+\underset{B}{\underbrace{\left[\begin{array}{cc} B_{11} & 0\\ B_{21} & B_{22} \end{array}\right]}}\left[\begin{array}{c} u_{1}\\ u_{2} \end{array}\right]+\left[\begin{array}{c} w_{1}\\ w_{2} \end{array}\right]\\ \; & \left[\begin{array}{c} y_{1}\\ y_{2} \end{array}\right]=\underset{C}{\underbrace{\left[\begin{array}{cc} C_{11} & 0\\ C_{21} & C_{22} \end{array}\right]}}\left[\begin{array}{c} x_{1}\\ x_{2} \end{array}\right] \end{array}$$

Notice the two-UAV system model in Equation (3) can be described in this form. For generality, from here the word ’player’ is used instead of ’UAV’ until the optimal strategies are derived.

In the nested dynamics framework described above, $x_{i}$ and $u_{i}$ denote the state variable and control input of *i*-th player, respectively. Please note that the above equations imply that the first player’s state and decision affects the second player, while the second player’s does not affect the first player. Also, the first player’s sensor measurement is available to the second player (via communication networks or by direct measurements), so the second player can use the first player’s information for its control ($u_{2}$ depends on $y_{2}$ and $y_{1}$), while the first player is not able to use the second player’s measurements ($u_{1}$ depends on $y_{1}$ only). Here, we assume that each player’s sensor measures its state directly which indicates that C=I, and therefore $y_{i}=x_{i}$ for i=1,2.

Define the random variables of the initial states which are mutually independent with the following known probability density functions:

(5) $$\begin{array}{cc} x_{1}\left(0\right) & ∼N\left({\bar{x}}_{1}\left(0\right),X_{1}\left(0\right)\right)\\ x_{2}\left(0\right) & ∼N\left({\bar{x}}_{2}\left(0\right),X_{2}\left(0\right)\right) \end{array}$$

and the process noises are assumed to be stationary zero-mean Gaussian which are characterized by the following covariance matrices:

(6) $$\begin{array}{cc} E\left[w_{1}\left(t\right)w_{1}\left(\tau \right)\right] & =W_{1}\left(t\right)\delta \left(t-\tau \right)\\ E\left[w_{2}\left(t\right)w_{2}\left(\tau \right)\right] & =W_{2}\left(t\right)\delta \left(t-\tau \right)\\ E\left[w_{1}\left(t\right)w_{2}\left(\tau \right)\right] & =0 \end{array}$$

The set of available information for each player is defined as follows:

(7) $$\begin{array}{cc} z_{1} & =\left\{y_{1}\right\}\\ z_{2} & =\left\{y_{1},y_{2}\right\} \end{array}$$

which gives the control strategies as:

(8) $$\begin{array}{cc} u_{1} & =f_{1}\left(z_{1}\right)\\ u_{2} & =f_{2}\left(z_{2}\right) \end{array}$$

where $f_{i}\left(z_{i}\right)$ is a function or a dynamical system that describes the controller of the *i*-th player.

The performance index in the finite-horizon quadratic form which couples the states $x_{1}$ and $x_{2}$ is defined as follows:

(9) $$J=E\left[x_{t_{f}}^{T}Hx_{t_{f}}+\int_{t_{0}}^{t_{f}}\left(x{\left(t\right)}^{T}Qx\left(t\right)+u{\left(t\right)}^{T}Ru\left(t\right)\right)dt\right]$$

where the weighting parameters *H* and *Q* are positive semidefinite and *R* is positive definite as follows:

$$\begin{array}{c} H=\left[\begin{array}{cc} H_{11} & H_{21}^{T}\\ H_{21} & H_{22} \end{array}\right],\;Q=\left[\begin{array}{cc} Q_{11} & Q_{21}^{T}\\ Q_{21} & Q_{22} \end{array}\right],\;R=\left[\begin{array}{cc} R_{11} & 0\\ 0 & R_{22} \end{array}\right] \end{array}$$

Then the optimization problem describing the cooperation of the two players can be stated as follows:

**Problem 1.**  *(Two player LQR) For the following two-player nested dynamical systems model,*

$$\begin{array}{cc} \; & \left[\begin{array}{c} {\dot{x}}_{1}\\ {\dot{x}}_{2} \end{array}\right]=\left[\begin{array}{cc} A_{11} & 0\\ A_{21} & A_{22} \end{array}\right]\left[\begin{array}{c} x_{1}\\ x_{2} \end{array}\right]+\left[\begin{array}{cc} B_{11} & 0\\ B_{21} & B_{22} \end{array}\right]\left[\begin{array}{c} u_{1}\\ u_{2} \end{array}\right]+\left[\begin{array}{c} w_{1}\\ w_{2} \end{array}\right]\\ \; & \left[\begin{array}{c} y_{1}\\ y_{2} \end{array}\right]=\left[\begin{array}{cc} I & 0\\ 0 & I \end{array}\right]\left[\begin{array}{c} x_{1}\\ x_{2} \end{array}\right] \end{array}$$

*and find the optimal strategies $u_{1}$ and $u_{2}$ that minimize the finite-horizon quadratic cost*

$$J=E\left[x_{t_{f}}^{T}Hx_{t_{f}}+\int_{t_{0}}^{t_{f}}\left(x{\left(t\right)}^{T}Qx\left(t\right)+u{\left(t\right)}^{T}Ru\left(t\right)\right)dt\right]$$

*where $u_{1}\left(t\right)$ is a function of $y_{1}\left(\tau \right)$ for 0≤τ≤t, and $u_{2}\left(t\right)$ is a function of $y_{1}\left(\tau \right)$ and $y_{2}\left(\tau \right)$ for 0≤τ≤t.*

### 2.3. State Separation

The centralized controller for linear quadratic regulation problem is well known that the full-state feedback is the optimal strategy, i.e., every player needs access to every information. However, in decentralized control problem it is impracticable because of the information asymmetry; player 1 does not have measurement information on player 2. Therefore, the state-separation principle is proposed which separates player 2’s state ($x_{2}$) into two parts: (1) player 1’s best estimate on player 2’s state ($x_{2|1}$), and (2) the remainder ($x_{2}-x_{2|1}=\Delta x_{2}$, i.e., the estimation error). The accessible information with information set $z_{i}$ can be written to the conditional estimation $E\left[x|z_{i}\right]$ which are as follows:

- For player 1:
  (10) $$E\left[x|z_{1}\right]:=\left[\begin{array}{c} x_{1|1}\\ x_{2|1} \end{array}\right]$$
  where $E\left[x_{1|1}\right]=E\left[x_{1}\right]$.
- For player 2:
  (11) $$E\left[x|z_{2}\right]:=\left[\begin{array}{c} x_{1|2}\\ x_{2|2} \end{array}\right]$$
  where $E\left[x_{1|2}\right]=E\left[x_{1}\right]$ and $E\left[x_{2|2}\right]=E\left[x_{2}\right]$.

The subscript j|i indicates the state of player *j* estimated by player *i*. Notice that player 1 must estimate the state of player 2 which is defined by $x_{2|1}$, and the best estimation is given by the dynamical propagation

(12) $$\begin{array}{c} {\dot{x}}_{2|1}=A_{21}x_{1}+A_{22}x_{2|1}+B_{21}u_{1}+B_{22}u_{2|1} \end{array}$$

which player 1 can compute only using player 1’s measurement information and player 2’s control strategy (it is assumed that the collaborator’s control strategy is known to each other). The definition of the estimation error of $x_{2|1}$ and the corresponding control input $u_{2|1}=f_{2}\left(x_{2|1}\right)$ are as follows:

(13) $$\begin{array}{c} \Delta x_{2}=x_{2}-x_{2|1}\\ \Delta u_{2}=u_{2}-u_{2|1} \end{array}$$

where the estimation error dynamics is given by:

(14) $$\begin{array}{cc} \Delta {\dot{x}}_{2} & ={\dot{x}}_{2}-{\dot{x}}_{2|1}\\ & =A_{22}\Delta x+B_{22}\Delta u_{2}+w_{2} \end{array}$$

Substituting (13) into the system model in Equation (4), the system can be rewritten with the newly defined separated state $x_{\mathrm{dec}}$ as follows:

(15) $$x_{\mathrm{dec}}:=\left[\begin{array}{c} x_{1}\\ x_{2|1}\\ \Delta x_{2} \end{array}\right]$$

(16) $$\begin{array}{c} {\dot{x}}_{\mathrm{dec}}=\underset{A_{\mathrm{dec}}}{\underbrace{\left[\begin{array}{ccc} A_{11} & 0 & 0\\ A_{21} & A_{22} & 0\\ 0 & 0 & A_{22} \end{array}\right]}}x_{\mathrm{dec}}+\underset{B_{\mathrm{dec}}}{\underbrace{\left[\begin{array}{ccc} B_{11} & 0 & 0\\ B_{21} & B_{22} & 0\\ 0 & 0 & B_{22} \end{array}\right]}}\left[\begin{array}{c} u_{1}\\ u_{2|1}\\ \Delta u_{2} \end{array}\right]+\left[\begin{array}{c} w_{1}\\ 0\\ w_{2} \end{array}\right] \end{array}$$

or separately

(17) $$\begin{array}{c} \left[\begin{array}{c} \dot{x_{1}}\\ {\dot{x}}_{2|1} \end{array}\right]=\left[\begin{array}{cc} A_{11} & 0\\ A_{21} & A_{22} \end{array}\right]\left[\begin{array}{c} x_{1}\\ x_{2|1} \end{array}\right]+\left[\begin{array}{cc} B_{11} & 0\\ B_{21} & B_{22} \end{array}\right]\left[\begin{array}{c} u_{1}\\ u_{2|1} \end{array}\right]+\left[\begin{array}{c} w_{1}\\ 0 \end{array}\right] \end{array}$$

and

(18) $$\begin{array}{c} \Delta {\dot{x}}_{2}=A_{22}\Delta x_{2}+B_{22}\Delta u_{2}+w_{2} \end{array}$$

### 2.4. Uncorrelated Variance Propagation

In this subsection, it is shown that the variances of state accessible to player 1 and the estimation error are propagated independently, and it is used to derive two independent subproblems that give insight of the optimal strategy. The initial probability density functions in Equation (5) are assumed to be known to each other, where the initial probability density functions of $x_{2|1}$ and $\Delta x_{2}$ are as follows:

(19) $$\begin{array}{cc} x_{2|1}\left(0\right) & ∼N({\bar{x}}_{2}\left(0\right),X_{2}\left(0\right))\\ \Delta x_{2}\left(0\right) & ∼N(0,2X_{2}\left(0\right)) \end{array}$$

giving the structured covariance matrix of $x_{\mathrm{dec}}\left(0\right)$ as follows:

(20) $$P\left(0\right)=\left[\begin{array}{ccc} P_{11}\left(0\right) & 0 & 0\\ 0 & P_{22}\left(0\right) & 0\\ 0 & 0 & P_{33}\left(0\right) \end{array}\right]$$

Consider a block 3×3 covariance matrix H which is structured as follows:

(21) H=−−.H1−−.00−H2−

where $H_{1}$ is block 2×2 (size compatible with *A*) and $H_{2}$ is block 1×1 (size compatible with $A_{22})$. Suppose an arbitrary feedback gain matrix $C_{\mathrm{dec}}$ which has the same structure as H, then *P* propagates with the following Lyapunov equation:

(22) $$\begin{array}{c} \dot{P}=\left(A_{\mathrm{dec}}-B_{\mathrm{dec}}C_{\mathrm{dec}}\right)P+P{\left(A_{\mathrm{dec}}-B_{\mathrm{dec}}C_{\mathrm{dec}}\right)}^{T}+Q \end{array}$$

where $A_{\mathrm{dec}}$ and $B_{\mathrm{dec}}$ are the system and input matrix in Equation (16). In addition, the autocorrelation of process noise matrix, *Q*, is:

$$\begin{array}{c} Q=\left[\begin{array}{ccc} W_{1} & 0 & 0\\ 0 & 0 & 0\\ 0 & 0 & W_{2} \end{array}\right] \end{array}$$

hence $A_{\mathrm{dec}}$, $B_{\mathrm{dec}}$, $C_{\mathrm{dec}}$, and *Q* have the same structures as H, and consequently the covariance matrix P(t) also follows the same structure as below:

(23) P(t)=P11(t)P12(t)P21(t)P22(t)000  0P33(t)

Therefore, $\left\{x_{1},x_{2|1}\right\}$ and $\left\{\Delta x_{2}\right\}$ are uncorrelated and independent if $C_{\mathrm{dec}}$ follows the same structure as H.

### 2.5. Cost Separation

The cost function in Equation (9) can be rewritten by substituting the state with the separated state in Equation (15) which yields

(24) $$J=E\left[x_{\mathrm{dec}}^{T}\left(t_{f}\right)H_{\mathrm{dec}}x_{\mathrm{dec}}\left(t_{f}\right)+\int_{t_{0}}^{t_{f}}\left(x_{\mathrm{dec}}^{T}\left(t\right)Q_{\mathrm{dec}}x_{\mathrm{dec}}\left(t\right)+u_{\mathrm{dec}}^{T}\left(t\right)R_{\mathrm{dec}}u_{\mathrm{dec}}\left(t\right)\right)dt\right]$$

where the state variables and the control variables are given by $x_{\mathrm{dec}}$=${\left[\begin{array}{ccc} x_{1}^{T} & x_{2|1}^{T} & \Delta x_{2}^{T} \end{array}\right]}^{T}$ and $u_{\mathrm{dec}}={\left[\begin{array}{ccc} u_{1}^{T} & u_{2|1}^{T} & \Delta u_{2}^{T} \end{array}\right]}^{T}$, and $H_{\mathrm{dec}}$, $Q_{\mathrm{dec}}$, $R_{\mathrm{dec}}$ are given by:

$$\begin{array}{c} H_{\mathrm{dec}}=\left[\begin{array}{ccc} H_{11} & H_{21}^{T} & H_{21}^{T}\\ H_{21} & H_{22} & H_{22}\\ H_{21} & H_{22} & H_{22} \end{array}\right]\;Q_{\mathrm{dec}}=\left[\begin{array}{ccc} Q_{11} & Q_{21}^{T} & Q_{21}^{T}\\ Q_{21} & Q_{22} & Q_{22}\\ Q_{21} & Q_{22} & Q_{22} \end{array}\right]\;R_{\mathrm{dec}}=\left[\begin{array}{ccc} R_{11} & 0 & 0\\ 0 & R_{22} & R_{22}\\ 0 & R_{22} & R_{22} \end{array}\right] \end{array}$$

which helps to rewrite Problem 1 by using the following state-separated model.

**Problem 2.**  *(Decentralized two player LQR) Consider the separated system model*

$$\begin{array}{cc} {\dot{x}}_{\mathrm{dec}} & =\left[\begin{array}{ccc} A_{11} & 0 & 0\\ A_{21} & A_{22} & 0\\ 0 & 0 & A_{22} \end{array}\right]x_{\mathrm{dec}}+\left[\begin{array}{ccc} B_{11} & 0 & 0\\ B_{21} & B_{22} & 0\\ 0 & 0 & B_{22} \end{array}\right]\left[\begin{array}{c} u_{1}\\ u_{2|1}\\ \Delta u_{2} \end{array}\right]+\left[\begin{array}{c} w_{1}\\ 0\\ w_{2} \end{array}\right]\\ \left[\begin{array}{c} y_{1}\\ y_{2} \end{array}\right] & =\left[\begin{array}{ccc} 1 & 0 & 0\\ 0 & 1 & 1 \end{array}\right]x_{\mathrm{dec}} \end{array}$$

*and find the optimal strategies $u_{\mathrm{dec}}={\left[\begin{array}{ccc} u_{1}^{T} & u_{2|1}^{T} & \Delta u_{2}^{T} \end{array}\right]}^{T}$ that minimize the cost*

$$J=E\left[x_{\mathrm{dec}}^{T}\left(t_{f}\right)H_{\mathrm{dec}}x_{\mathrm{dec}}\left(t_{f}\right)+\int_{t_{0}}^{t_{f}}\left(x_{\mathrm{dec}}^{T}\left(t\right)Q_{\mathrm{dec}}x_{\mathrm{dec}}\left(t\right)+u_{\mathrm{dec}}^{T}\left(t\right)R_{\mathrm{dec}}u_{\mathrm{dec}}\left(t\right)\right)dt\right]$$

*where $u_{1}\left(t\right)$ and $u_{2|1}\left(t\right)$ are functions of $y_{1}\left(\tau \right)$ for 0≤τ≤t, and $\Delta u_{2}\left(t\right)$ is a function of $y_{1}\left(\tau \right)$ and $y_{2}\left(\tau \right)$ for 0≤τ≤t.*

Based on the fact that $\left\{x_{1},x_{2|1}\right\}$ and $\left\{\Delta x_{2}\right\}$ are independent, Equation (24) can be separated into three parts following the structures of H.

(25) $$\begin{array}{c} J=J_{1}+J_{2}+J_{3} \end{array}$$

- Part 1:
  (26) $$\begin{array}{c} J_{1}=E[{\left[\begin{array}{c} x_{1}\\ x_{2|1} \end{array}\right]}_{t_{f}}^{T}\left[\begin{array}{cc} H_{11} & H_{21}^{T}\\ H_{21} & H_{22} \end{array}\right]{\left[\begin{array}{c} x_{1}\\ x_{2|1} \end{array}\right]}_{t_{f}}\\ +\int_{t_{0}}^{t_{f}}\left({\left[\begin{array}{c} x_{1}\\ x_{2|1} \end{array}\right]}^{T}\left[\begin{array}{cc} Q_{11} & Q_{21}^{T}\\ Q_{21} & Q_{22} \end{array}\right]\left[\begin{array}{c} x_{1}\\ x_{2|1} \end{array}\right]+{\left[\begin{array}{c} u_{1}\\ u_{2|1} \end{array}\right]}^{T}\left[\begin{array}{cc} R_{11} & 0\\ 0 & R_{22} \end{array}\right]\left[\begin{array}{c} u_{1}\\ u_{2|1} \end{array}\right]\right)dt] \end{array}$$
- Part 2:
  (27) $$J_{2}=E\left[\Delta x_{2}^{T}\left(t_{f}\right)H_{22}\Delta x_{2}\left(t_{f}\right)+\int_{t_{0}}^{t_{f}}\left(\Delta x_{2}^{T}Q_{22}\Delta x_{2}+\Delta u_{2}^{T}R_{22}\Delta u_{2}\right)dt\right]$$
- Part 3:
  (28) $$\begin{array}{c} J_{3}=E[{\left[\begin{array}{c} x_{1}\\ x_{2|1}\\ \Delta x_{2} \end{array}\right]}_{t_{f}}^{T}\left[\begin{array}{ccc} 0 & 0 & H_{21}^{T}\\ 0 & 0 & H_{22}\\ H_{21} & H_{22} & 0 \end{array}\right]{\left[\begin{array}{c} x_{1}\\ x_{2|1}\\ \Delta x_{2} \end{array}\right]}_{t_{f}}\\ +\int_{t_{0}}^{t_{f}}\left({\left[\begin{array}{c} x_{1}\\ x_{2|1}\\ \Delta x_{2} \end{array}\right]}^{T}\left[\begin{array}{ccc} 0 & 0 & Q_{21}^{T}\\ 0 & 0 & Q_{22}\\ Q_{21} & Q_{22} & 0 \end{array}\right]\left[\begin{array}{c} x_{1}\\ x_{2|1}\\ \Delta x_{2} \end{array}\right]+{\left[\begin{array}{c} u_{1}\\ u_{2|1}\\ \Delta u_{2} \end{array}\right]}^{T}\left[\begin{array}{ccc} 0 & 0 & 0\\ 0 & 0 & R_{22}\\ 0 & R_{22} & 0 \end{array}\right]\left[\begin{array}{c} u_{1}\\ u_{2|1}\\ \Delta u_{2} \end{array}\right]\right)dt] \end{array}$$

The cost $J_{1}$ and $J_{2}$ represent the performance index of the strategies by $\left\{x_{1},x_{2|1}\right\}$ and $\left\{\Delta x_{2}\right\}$ respectively, while $J_{3}$ describes the cross-coupling of $\left\{x_{1},x_{2|1}\right\}$ and $\left\{\Delta x_{2}\right\}$. Suppose the weighting matrix H_dec_ and Q_dec_ are symmetric, then $J_{3}$ can be simplified as

(29) $$J_{3}=E\left[2{\left[\begin{array}{cc} x_{1} & x_{2|1} \end{array}\right]}_{t_{f}}\left[\begin{array}{c} H_{21}\\ H_{22} \end{array}\right]{\left[\begin{array}{c} \Delta x_{2} \end{array}\right]}_{t_{f}}+2\int_{t_{0}}^{t_{f}}\left(\left[\begin{array}{cc} x_{1} & x_{2|1} \end{array}\right]\left[\begin{array}{c} Q_{21}\\ Q_{22} \end{array}\right]\left[\begin{array}{c} \Delta x_{2} \end{array}\right]+u_{2|1}R_{22}\Delta u_{2}\right)dt\right]$$

where $u_{2|1}$ and $\Delta u_{2}$ are linear in $x_{2|1}$ and $\Delta x_{2}$ respectively. Since $\left\{x_{1},x_{2|1}\right\}$ and $\left\{\Delta x_{2}\right\}$ are independent random variables with the initial probability density functions given in Equations (5) and (19) where their variance propagate with the Lyapunov equation in Equation (22), the expected values of the linear combinations of $\left\{x_{1},x_{2|1}\right\}$ and $\left\{\Delta x_{2}\right\}$ can be derived as follows:

(30) $$E\left[x_{1}\Delta x_{2}\right]=E\left[x_{1}\right]E\left[\Delta x_{2}\right]=0$$

and

(31) $$E\left[x_{2|1}\Delta x_{2}\right]=E\left[x_{2|1}\right]E\left[\Delta x_{2}\right]=0$$

Therefore, $J_{1}$ and $J_{2}$ are functions of independent variables and consequently $J_{3}=0$. Then the minimum of *J* is given by the summation of the minimums of $J_{1}$ and $J_{2}$ as follows:

(32) $$\begin{array}{c} \min_{u_{\mathrm{dec}}}J=\min_{u_{1},u_{2|1}}J_{1}+\min_{\Delta u_{2}}J_{2} \end{array}$$

Now the optimal solution of Problem 2 can be obtained by solving two independent subproblems. The solution of each subproblem can be obtained by each player, the leader and the follower.

**Subproblem 1.**  *(Leader problem) Consider the separated model for the leader*

$$\begin{array}{c} \left[\begin{array}{c} \dot{x_{1}}\\ {\dot{x}}_{2|1} \end{array}\right]=\left[\begin{array}{cc} A_{11} & 0\\ A_{21} & A_{22} \end{array}\right]\left[\begin{array}{c} x_{1}\\ x_{2|1} \end{array}\right]+\left[\begin{array}{cc} B_{11} & 0\\ B_{21} & B_{22} \end{array}\right]\left[\begin{array}{c} u_{1}\\ u_{2|1} \end{array}\right]+\left[\begin{array}{c} w_{1}\\ 0 \end{array}\right] \end{array}$$

*where the accessible information for the leader is limited to*

$$y_{1}=x_{1}$$

*Find the optimal strategies $u_{1}$ and $u_{2|1}$ that minimize the cost*

$$\begin{array}{c} J_{1}=E[{\left[\begin{array}{c} x_{1}\\ x_{2|1} \end{array}\right]}_{t_{f}}^{T}\left[\begin{array}{cc} H_{11} & H_{21}^{T}\\ H_{21} & H_{22} \end{array}\right]{\left[\begin{array}{c} x_{1}\\ x_{2|1} \end{array}\right]}_{t_{f}}\\ +\int_{t_{0}}^{t_{f}}\left({\left[\begin{array}{c} x_{1}\\ x_{2|1} \end{array}\right]}^{T}\left[\begin{array}{cc} Q_{11} & Q_{21}^{T}\\ Q_{21} & Q_{22} \end{array}\right]\left[\begin{array}{c} x_{1}\\ x_{2|1} \end{array}\right]+{\left[\begin{array}{c} u_{1}\\ u_{2|1} \end{array}\right]}^{T}\left[\begin{array}{cc} R_{11} & 0\\ 0 & R_{22} \end{array}\right]\left[\begin{array}{c} u_{1}\\ u_{2|1} \end{array}\right]\right)dt] \end{array}$$

*where $u_{1}\left(t\right)$ and $u_{2|1}\left(t\right)$ are functions of $y_{1}\left(\tau \right)$ for 0≤τ≤t.*

**Subproblem 2.**  *(Follower problem) Consider the separated model for the follower*

$$\begin{array}{c} \Delta {\dot{x}}_{2}=A_{22}\Delta x_{2}+B_{22}\Delta u_{2}+w_{2} \end{array}$$

*where the follower is accessible to every information*

$$\left[\begin{array}{c} y_{1}\\ y_{2} \end{array}\right]=\left[\begin{array}{cc} I & 0\\ 0 & I \end{array}\right]\left[\begin{array}{c} x_{1}\\ x_{2} \end{array}\right]$$

*Find the optimal strategy $\Delta u_{2}$ that minimizes the cost*

$$J_{2}=E\left[\Delta x_{2}^{T}\left(t_{f}\right)H_{22}\Delta x_{2}\left(t_{f}\right)+\int_{t_{0}}^{t_{f}}\left(\Delta x_{2}^{T}Q_{22}\Delta x_{2}+\Delta u_{2}^{T}R_{22}\Delta u_{2}\right)dt\right]$$

*where $\Delta u_{2}\left(t\right)$ is a function of $y_{1}\left(\tau \right)$ and $y_{2}\left(\tau \right)$ for 0≤τ≤t.*

## 3. Main Results

In this section, the explicit optimal control strategy of the Problem 1 is obtained. Then based on the solution, the centralized and decentralized optimal two-agent cooperative guidance laws are derived.

### 3.1. Decentralized Two-Player Optimal Controller

**Lemma 1.**  *Consider the Subproblem 1 and suppose that there exists a stabilizing solution X for the Riccati equation*

(33) $$\begin{array}{c} \dot{X}+A^{T}X+XA+Q-XBR^{-1}B^{T}X=0 \end{array}$$

*with the terminal condition*

(34) $$\begin{array}{c} X(t_{f})=H \end{array}$$

*where A,B are the system and input matrix in Equation (17) and H is the terminal weighting matrix defined in Equation (26).*

*Then the optimal strategies for Subproblem 1 are as follows:*

(35) $$\begin{array}{cc} \left[\begin{array}{c} u_{1}\\ u_{2|1} \end{array}\right] & =K\left[\begin{array}{c} x_{1}\\ x_{2|1} \end{array}\right]=\left[\begin{array}{cc} K_{11} & K_{12}\\ K_{21} & K_{22} \end{array}\right]\left[\begin{array}{c} x_{1}\\ x_{2|1} \end{array}\right] \end{array}$$

*where*

(36) $$\begin{array}{c} K=-R^{-1}B^{T}X \end{array}$$

*and $x_{2|1}$ evolves with the following dynamics:*

$${\dot{x}}_{2|1}=A_{21}x_{1}+A_{22}x_{2|1}+B_{21}u_{1}+B_{22}u_{2|1}$$

**Lemma 2.**  *Consider the Subproblem 2 and suppose that there exists a stabilizing solution Y for the Riccati equation*

(37) $$\begin{array}{c} \dot{Y}+A_{22}^{T}Y+YA_{22}+Q_{22}-YB_{22}R_{22}^{-1}B_{22}^{T}Y=0 \end{array}$$

*with the terminal condition*

(38) $$\begin{array}{c} Y\left(t_{f}\right)=H_{22} \end{array}$$

*Then the optimal strategy for Subproblem 2 is as follows:*

(39) $$\begin{array}{c} \Delta u_{2}=L\Delta x_{2} \end{array}$$

*where*

(40) $$\begin{array}{c} L=-R_{22}^{-1}B_{22}^{T}Y \end{array}$$

**Proof.**  (for both Lemma 1 and Lemma 2) For Subproblems 1 and 2, all the necessary information is accessible to each player as follows:

(41) $$\begin{array}{cc} \left\{x_{1},x_{2|1}\right\} & =x|z_{1} \end{array}$$

(42) $$\begin{array}{cc} \left\{x_{1},x_{2|1},\Delta x_{2}\right\} & =x|z_{2} \end{array}$$

therefore, each subproblem is a centralized problem with full information, for which the optimal strategy is the well-known full-state feedback obtained by solving the Riccati equations in Equations (33) and (37). For more technical details including the solution uniqueness issues, one may refer to [33] or [35].

**Theorem 1.**  *The optimal strategies for Problem 1 are*

(43) $$\begin{array}{c} \left[\begin{array}{c} u_{1}\\ u_{2} \end{array}\right]=K\left[\begin{array}{c} x_{1}\\ x_{2|1} \end{array}\right]+\left[\begin{array}{c} 0\\ L \end{array}\right]\Delta x_{2} \end{array}$$

*or*

(44) $$\begin{array}{c} \left[\begin{array}{c} u_{1}\\ u_{2} \end{array}\right]=\left[\begin{array}{cc} K_{11} & K_{12}\\ K_{21} & K_{22} \end{array}\right]\left[\begin{array}{c} x_{1}\\ x_{2|1} \end{array}\right]+\left[\begin{array}{c} 0\\ L \end{array}\right]\left(x_{2}-x_{2|1}\right) \end{array}$$

*where K and L are obtained from Equations (36) and (40), and the state prediction $x_{2|1}$ is obtained from*

$${\dot{x}}_{2|1}=A_{21}x_{1}+A_{22}x_{2|1}+B_{21}u_{1}+B_{22}u_{2|1}$$

**Proof.**  The proof follows from Lemma 1, Lemma 2 and the definition of $\Delta x_{2}$ and $\Delta u_{2}$ as follows:

$$\begin{array}{c} \left[\begin{array}{c} u_{1}\\ u_{2} \end{array}\right]=\left[\begin{array}{c} u_{1}\\ u_{2|1} \end{array}\right]+\left[\begin{array}{c} 0\\ \Delta u_{2} \end{array}\right] \end{array}$$

which achieves the minimum cost given in Equation (32).

### 3.2. Optimal Cooperative Guidance Laws for Two UAVs Relative Approach Angle Control

In this section, the main results in Theorem 1 are used for deriving the optimal cooperative guidance solutions for two UAVs under information constraints with the relative approach angle constraints. The solutions are expressed in terms of the line-of-sight parameters, so that they can be easily understood by aerospace guidance communities and can be efficiently implemented on conventional guidance computers.

#### 3.2.1. Centralized Solution with Full Information Sharing

For controlling the relative approach angle between two UAVs, a linear quadratic problem with the following cost function is considered:

(45) $$\min_{u}\;J=E\left[\frac{b}{2}\left(\zeta_{1}^{2}\left(t_{f}\right)+\zeta_{2}^{2}\left(t_{f}\right)\right)+\frac{c}{2}{\left({\dot{\zeta }}_{1}\left(t_{f}\right)-\alpha {\dot{\zeta }}_{2}\left(t_{f}\right)-r\right)}^{2}+\frac{1}{2}\int_{t_{0}}^{t_{f}}\left(a_{1}^{2}\left(t\right)+a_{2}^{2}\left(t\right)\right)dt\right]$$

where the notations follow from Figure 1, and the positive real number *b* and *c* are the penalties imposed on the UAVs’ miss distance and the approach angle error, respectively. The parameter *r* above represents the reference value inducing the terminal relative approach angle. The flight-path angle of each UAV is related to $V_{i}$ and ${\dot{\zeta }}_{i}$ as follows:

(46) $$\gamma_{i}=\sin^{-1}\frac{{\dot{\zeta }}_{{}_{i}}}{V_{{}_{i}}}≃\frac{{\dot{\zeta }}_{i}}{V_{i}}$$

and the parameter α is the ratio of $V_{1}$ and $V_{2}$. Without loss of generality, it is assumed that $V_{{}_{2}}$ is not smaller than $V_{1}$, hence:

(47) $$\alpha =\frac{V_{1}}{V_{2}}\leq 1$$

The relative approach angle of two UAVs, $\gamma_{\mathrm{rel}}$, which is required to be $\gamma_{\mathrm{rel}}\to \theta_{\mathrm{ref}}$ at the terminal time, can be expressed as follows:

(48) $$\begin{array}{c} \gamma_{\mathrm{rel}}=\gamma_{1}\left(t_{f}\right)-\gamma_{2}\left(t_{f}\right)≃\frac{{\dot{\zeta }}_{1}\left(t_{f}\right)}{V_{1}\left(t_{f}\right)}-\frac{{\dot{\zeta }}_{2}\left(t_{f}\right)}{V_{2}\left(t_{f}\right)} \end{array}$$

To obtain the optimal control input that minimizes Equation (45), the Hamiltonian is defined as follows:

(49) $$H=L+\nu^{T}f$$

where $L=\frac{1}{2}\left(a_{1}^{2}\left(t\right)+a_{2}^{2}\left(t\right)\right)$ and f=Ax+Bu, with the definitions of *x* and *u* from Equation (3). Hereafter in this subsection, we follow the same definitions for *x* and *u*. The Lagrangian multiplier ν is a column vector which consists of $\nu_{1}\left(t\right),\nu_{2}\left(t\right),\nu_{3}\left(t\right)$ and $\nu_{4}\left(t\right)$, and the first necessary conditions for optimality are the following costate equations:

(50) $$\begin{array}{c} \frac{\partial H}{\partial x}=-\dot{\nu }\\ {\dot{\nu }}_{1}=0,\;{\dot{\nu }}_{2}=-\nu_{1},\;{\dot{\nu }}_{3}=0,\;{\dot{\nu }}_{4}=-\nu_{3},\;u_{1}=-\nu_{2},\;u_{2}=-\nu_{4} \end{array}$$

with the terminal conditions as follows:

(51) $$\begin{array}{c} \nu_{1}\left(t_{f}\right)=b\zeta_{1}\left(t_{f}\right),\;\nu_{2}\left(t_{f}\right)=c\left({\dot{\zeta }}_{1}\left(t_{f}\right)-\alpha {\dot{\zeta }}_{2}\left(t_{f}\right)-r\right)\\ \nu_{3}\left(t_{f}\right)=b\zeta_{2}\left(t_{f}\right),\;\nu_{4}\left(t_{f}\right)=-c\alpha \left({\dot{\zeta }}_{1}\left(t_{f}\right)-\alpha {\dot{\zeta }}_{2}\left(t_{f}\right)-r\right) \end{array}$$

thus, the dynamic equations of the costates are as follows:

(52) $$\begin{array}{c} \nu_{1}\left(t\right)=b\zeta_{1}\left(t_{f}\right),\;\nu_{2}\left(t\right)=b\zeta_{1}\left(t_{f}\right)\left(t_{f}-t\right)+c\left({\dot{\zeta }}_{1}\left(t_{f}\right)-\alpha {\dot{\zeta }}_{2}\left(t_{f}\right)-r\right)\\ \nu_{3}\left(t\right)=b\zeta_{2}\left(t_{f}\right),\;\nu_{4}\left(t\right)=b\zeta_{2}\left(t_{f}\right)\left(t_{f}-t\right)-c\alpha \left({\dot{\zeta }}_{1}\left(t_{f}\right)-\alpha {\dot{\zeta }}_{2}\left(t_{f}\right)-r\right) \end{array}$$

The second necessary condition for optimality is as follows:

(53) $$\frac{\partial H}{\partial a}=0$$

which yields

(54) $$\begin{array}{c} \begin{array}{cc} a_{1}\left(t\right) & =-\left[b\zeta_{1}\left(t_{f}\right)\left(t_{f}-t\right)+c\left({\dot{\zeta }}_{1}\left(t_{f}\right)-\alpha {\dot{\zeta }}_{2}\left(t_{f}\right)-r\right)\right]\\ a_{2}\left(t\right) & =-\left[b\zeta_{2}\left(t_{f}\right)\left(t_{f}-t\right)-c\alpha \left({\dot{\zeta }}_{1}\left(t_{f}\right)-\alpha {\dot{\zeta }}_{2}\left(t_{f}\right)-r\right)\right] \end{array} \end{array}$$

Substituting the above optimal control solutions to the linear system description in Equation (3) and integrating yields:

(55) $$\begin{array}{c} \begin{array}{cc} \zeta_{1}\left(t\right) & =\left[\frac{1}{2}b\left(\frac{t^{3}}{3}-\frac{t_{0}^{3}}{3}\right)-\frac{1}{2}bt_{0}^{2}\left(t-t_{0}\right)\right]\zeta_{1}\left(t_{f}\right)-\frac{1}{2}b{\left(t-t_{0}\right)}^{2}t_{f}\zeta_{1}\left(t_{f}\right)\\ \; & \;-\frac{1}{2}c{\left(t-t_{0}\right)}^{2}\left({\dot{\zeta }}_{1}\left(t_{f}\right)-\alpha {\dot{\zeta }}_{2}\left(t_{f}\right)-r\right)+{\dot{\zeta }}_{1}\left(t_{0}\right)\left(t-t_{0}\right)+\zeta_{1}\left(t_{0}\right)\\ {\dot{\zeta }}_{1}\left(t\right) & =\frac{1}{2}b\left(t^{2}-t_{0}^{2}\right)\zeta_{1}\left(t_{f}\right)-b\left(t-t_{0}\right)\zeta_{1}\left(t_{f}\right)t_{f}-c\left({\dot{\zeta }}_{1}\left(t_{f}\right)-\alpha {\dot{\zeta }}_{2}\left(t_{f}\right)-r\right)\left(t-t_{0}\right)+{\dot{\zeta }}_{1}\left(t_{0}\right)\\ \zeta_{2}\left(t\right) & =\left[\frac{1}{2}b\left(\frac{t^{3}}{3}-\frac{t_{0}^{3}}{2}\right)-\frac{1}{2}bt_{0}^{2}\left(t-t_{0}\right)\right]\zeta_{2}\left(t_{f}\right)-\frac{1}{2}b{\left(t-t_{0}\right)}^{2}t_{f}\zeta_{2}\left(t_{f}\right)\\ \; & \;+\frac{1}{2}c\alpha {\left(t-t_{0}\right)}^{2}\left({\dot{\zeta }}_{1}\left(t_{f}\right)-\alpha {\dot{\zeta }}_{2}\left(t_{f}\right)-r\right)+{\dot{\zeta }}_{2}\left(t_{0}\right)\left(t-t_{0}\right)+\zeta_{2}\left(t_{0}\right)\\ {\dot{\zeta }}_{2}\left(t\right) & =\frac{1}{2}b\left(t^{2}-t_{0}^{2}\right)\zeta_{2}\left(t_{f}\right)-b\left(t-t_{0}\right)\zeta_{2}\left(t_{f}\right)t_{f}+c\alpha \left({\dot{\zeta }}_{1}\left(t_{f}\right)-\alpha {\dot{\zeta }}_{2}\left(t_{f}\right)-r\right)\left(t-t_{0}\right)+{\dot{\zeta }}_{2}\left(t_{0}\right) \end{array} \end{array}$$

along with $\tau =t_{f}-t_{0}$ and $t=t_{f}$, Equation (55) can be expressed in terms of $t_{f}$ as follows:

(56) $$\begin{array}{c} \begin{array}{cc} \zeta_{1}\left(t_{f}\right) & =-\frac{1}{3}b\tau^{3}\zeta_{1}\left(t_{f}\right)-\frac{1}{2}c\tau^{2}\left({\dot{\zeta }}_{1}\left(t_{f}\right)-\alpha {\dot{\zeta }}_{2}\left(t_{f}\right)-r\right)+{\dot{\zeta }}_{1}\left(t_{0}\right)\tau +\zeta_{1}\left(t_{0}\right)\\ {\dot{\zeta }}_{1}\left(t_{f}\right) & =-\frac{1}{2}b\tau^{2}\zeta_{1}\left(t_{f}\right)-c\left({\dot{\zeta }}_{1}\left(t_{f}\right)-\alpha {\dot{\zeta }}_{2}\left(t_{f}\right)-r\right)\tau +{\dot{\zeta }}_{1}\left(t_{0}\right)\\ \zeta_{2}\left(t_{f}\right) & =-\frac{1}{3}b\tau^{3}\zeta_{2}\left(t_{f}\right)+\frac{1}{2}c\alpha \tau^{2}\left({\dot{\zeta }}_{1}\left(t_{f}\right)-\alpha {\dot{\zeta }}_{2}\left(t_{f}\right)-r\right)+{\dot{\zeta }}_{2}\left(t_{0}\right)\tau +\zeta_{2}\left(t_{0}\right)\\ {\dot{\zeta }}_{2}\left(t_{f}\right) & =-\frac{1}{2}b\tau^{2}\zeta_{2}\left(t_{f}\right)+c\alpha \left({\dot{\zeta }}_{1}\left(t_{f}\right)-\alpha {\dot{\zeta }}_{2}\left(t_{f}\right)-r\right)\tau +{\dot{\zeta }}_{2}\left(t_{0}\right) \end{array} \end{array}$$

Evaluating the above equation with the transition matrix $\Phi (t_{f},t_{0})$ and *r* gives the following linear relations:

(57) $$\begin{array}{c} x\left(t_{f}\right)=\Phi \left(t_{f},t_{0}\right)x\left(t_{0}\right)+\Psi r \end{array}$$

where

$$\begin{array}{cc} \Phi (t_{f},t_{0}) & ={\left(I-A^{\prime }\right)}^{-1}B^{\prime }\\ \Psi & ={\left(I-A^{\prime }\right)}^{-1}S \end{array}$$

and

(58) $$\begin{array}{c} A^{\prime }=\left[\begin{array}{cccc} -\frac{1}{3}b\tau^{3} & -\frac{1}{2}c\tau^{2} & 0 & \frac{1}{2}c\alpha \tau^{2}\\ -\frac{1}{2}b\tau^{2} & -c\tau & 0 & c\alpha \tau \\ 0 & \frac{1}{2}c\alpha \tau^{2} & -\frac{1}{3}b\tau^{3} & -\frac{1}{2}c\alpha^{2}\tau^{2}\\ 0 & c\alpha \tau & -\frac{1}{2}b\tau^{2} & -c\alpha^{2}\tau \end{array}\right],\;B^{\prime }=\left[\begin{array}{cccc} 1 & \tau & 0 & 0\\ 0 & 1 & 0 & 0\\ 0 & 0 & 1 & \tau \\ 0 & 0 & 0 & 1 \end{array}\right],\;S=\left[\begin{array}{c} \frac{1}{2}c\tau^{2}\\ c\tau \\ -\frac{1}{2}c\alpha \tau^{2}\\ -c\alpha \tau \end{array}\right] \end{array}$$

Then the resulting linear algebraic equations can be solved for the unknown terminal values $x(t_{f})$ and thus arrive at the expression for $u\left(t\right)={\left[\begin{array}{cc} a_{1}\left(t\right) & a_{2}\left(t\right) \end{array}\right]}^{T}$ by replacing the arbitrary $t_{0}$ by *t* as follows:

(59) $$\begin{array}{c} \begin{array}{cc} \left[\begin{array}{c} a_{1}\left(t\right)\\ a_{2}\left(t\right) \end{array}\right]\; & =-\left[\begin{array}{c} b\zeta_{1}\left(t_{f}\right)\tau +c\left({\dot{\zeta }}_{1}\left(t_{f}\right)-\alpha {\dot{\zeta }}_{2}\left(t_{f}\right)-r\right)\\ b\zeta_{2}\left(t_{f}\right)\tau -c\alpha \left({\dot{\zeta }}_{1}\left(t_{f}\right)-\alpha {\dot{\zeta }}_{2}\left(t_{f}\right)-r\right) \end{array}\right]\\ \; & =-\left[\begin{array}{cccc} b\tau & c & 0 & -c\alpha \\ 0 & -c\alpha & b\tau & c\alpha^{2} \end{array}\right]\left(\Phi \left(t_{f},t\right)x\left(t\right)+\Psi r\right)+\left[\begin{array}{c} c\\ -c\alpha \end{array}\right]r\\ \; & =-Kx(t)+Fr \end{array} \end{array}$$

where the optimal feedback gains $K_{ij}$ for i=1,2 and j=1,2,3,4 and the feed-forward gains $F_{k}$ for k=1,2 are obtained as follows:

(60) $$\begin{array}{c} K_{11}=\frac{\left(6b^{2}c+3b^{2}c\alpha^{2}\right)\tau^{5}+12b^{2}\tau^{4}+\left(18bc+36bc\alpha^{2}\right)\tau^{2}+36b\tau }{\left(b^{2}c\alpha^{2}+b^{2}c\right)\tau^{7}+\left(4b^{2}\right)\tau^{6}+\left(15bc\alpha^{2}+15bc\right)\tau^{4}+\left(24b\right)\tau^{3}+\left(36c\alpha^{2}+36c\right)\tau +36}\\ K_{12}=\frac{\left(4b^{2}c+3b^{2}c\alpha^{2}\right)\tau^{6}+12b^{2}\tau^{5}+\left(24bc+36bc\alpha^{2}\right)\tau^{3}+36b\tau^{2}+36c}{\left(b^{2}c\alpha^{2}+b^{2}c\right)\tau^{7}+\left(4b^{2}\right)\tau^{6}+\left(15bc\alpha^{2}+15bc\right)\tau^{4}+\left(24b\right)\tau^{3}+\left(36c\alpha^{2}+36c\right)\tau +36}\\ K_{13}=\frac{-3b^{2}c\alpha \tau^{5}+18bc\alpha \tau^{2}}{\left(b^{2}c\alpha^{2}+b^{2}c\right)\tau^{7}+\left(4b^{2}\right)\tau^{6}+\left(15bc\alpha^{2}+15bc\right)\tau^{4}+\left(24b\right)\tau^{3}+\left(36c\alpha^{2}+36c\right)\tau +36}\\ K_{14}=\frac{-b^{2}c\alpha \tau^{6}+12bc\alpha \tau^{3}-36c\alpha }{\left(b^{2}c\alpha^{2}+b^{2}c\right)\tau^{7}+\left(4b^{2}\right)\tau^{6}+\left(15bc\alpha^{2}+15bc\right)\tau^{4}+\left(24b\right)\tau^{3}+\left(36c\alpha^{2}+36c\right)\tau +36}\\ K_{21}=\frac{-3b^{2}c\alpha \tau^{5}+18bc\alpha \tau^{2}}{\left(b^{2}c\alpha^{2}+b^{2}c\right)\tau^{7}+\left(4b^{2}\right)\tau^{6}+\left(15bc\alpha^{2}+15bc\right)\tau^{4}+\left(24b\right)\tau^{3}+\left(36c\alpha^{2}+36c\right)\tau +36}\\ K_{22}=\frac{-b^{2}c\alpha \tau^{6}+12bc\alpha \tau^{3}-36c\alpha }{\left(b^{2}c\alpha^{2}+b^{2}c\right)\tau^{7}+\left(4b^{2}\right)\tau^{6}+\left(15bc\alpha^{2}+15bc\right)\tau^{4}+\left(24b\right)\tau^{3}+\left(36c\alpha^{2}+36c\right)\tau +36}\\ K_{23}=\frac{\left(3b^{2}c+6b^{2}c\alpha^{2}\right)\tau^{5}+12b^{2}\tau^{4}+\left(36bc+18bc\alpha^{2}\right)\tau^{2}+36b\tau }{\left(b^{2}c\alpha^{2}+b^{2}c\right)\tau^{7}+\left(4b^{2}\right)\tau^{6}+\left(15bc\alpha^{2}+15bc\right)\tau^{4}+\left(24b\right)\tau^{3}+\left(36c\alpha^{2}+36c\right)\tau +36}\\ K_{24}=\frac{\left(3b^{2}c+4ab^{2}c\alpha^{2}\right)\tau^{6}+12b^{2}\tau^{5}+\left(36bc+24bc\alpha^{2}\right)\tau^{3}+36b\tau^{2}+36c\alpha^{2}}{\left(b^{2}c\alpha^{2}+b^{2}c\right)\tau^{7}+\left(4b^{2}\right)\tau^{6}+\left(15bc\alpha^{2}+15bc\right)\tau^{4}+\left(24b\right)\tau^{3}+\left(36c\alpha^{2}+36c\right)\tau +36}\\ F_{1}=\frac{-2bc\tau^{3}+12c}{\left(bc+bc\alpha^{2}\right)\tau^{4}+4b\tau^{3}+\left(12c+12c\alpha^{2}\right)\tau +12}\\ F_{2}=\frac{12bc\alpha \tau^{3}-12c\alpha }{\left(bc+bc\alpha^{2}\right)\tau^{4}+4b\tau^{3}+\left(12c+12c\alpha^{2}\right)\tau +12} \end{array}$$

Taking the limit b,c→∞, for obtaining the zero-miss distance with the required relative approach angle, yields

(61) $$\begin{array}{cc} K_{11} & =\frac{3\alpha^{2}+6}{\left(1+\alpha^{2}\right)\tau^{2}},K_{12}=\frac{3\alpha^{2}+4}{(1+\alpha^{2})\tau },K_{13}=\frac{-3\alpha }{\left(1+\alpha^{2}\right)\tau^{2}},K_{14}=\frac{-\alpha }{(1+\alpha^{2})\tau },F_{1}=-\frac{2}{(1+\alpha^{2})\tau }\\ K_{21} & =\frac{-3\alpha }{\left(1+\alpha^{2}\right)\tau^{2}},K_{22}=\frac{-\alpha }{(1+\alpha^{2})\tau },K_{23}=\frac{6\alpha^{2}+3}{\left(1+\alpha^{2}\right)\tau^{2}},K_{24}=\frac{4\alpha^{2}+3}{(1+\alpha^{2})\tau },F_{2}=\frac{2\alpha }{(1+\alpha^{2})\tau } \end{array}$$

and then the optimal control strategies are obtained as follows:

(62) $$\begin{array}{c} \begin{array}{cc} a_{1}\left(t\right) & =-\frac{\left(3\alpha^{2}+4\right)\left(\zeta_{1}+{\dot{\zeta }}_{1}\tau \right)+2\zeta_{1}}{\left(1+\alpha^{2}\right)\tau^{2}}+\frac{\alpha \left(\zeta_{2}+{\dot{\zeta }}_{2}\tau \right)+2\alpha \zeta_{2}}{\left(1+\alpha^{2}\right)\tau^{2}}-\frac{2}{(1+\alpha^{2})\tau }\\ a_{2}\left(t\right) & =\frac{\alpha \left(\zeta_{1}+{\dot{\zeta }}_{1}\tau \right)+2\alpha \zeta_{1}}{\left(1+\alpha^{2}\right)\tau^{2}}-\frac{\left(4\alpha^{2}+3\right)\left(\zeta_{2}+{\dot{\zeta }}_{2}\tau \right)+2\alpha^{2}\zeta_{2}}{\left(1+\alpha^{2}\right)\tau^{2}}+\frac{2\alpha }{(1+\alpha^{2})\tau } \end{array} \end{array}$$

which can be rewritten in terms of the line-of-sight angles as follows:

(63) $$\begin{array}{c} \begin{array}{cc} a_{1}\left(t\right) & =\frac{1}{\left(1+\alpha^{2}\right)}\left[-V_{c,1}\left(\left(3\alpha^{2}+4\right){\dot{\lambda }}_{1}+\frac{2}{\tau }\lambda_{1}\right)+V_{c,2}\left(\alpha {\dot{\lambda }}_{2}+\frac{2\alpha }{\tau }\lambda_{2}\right)-\frac{2}{\tau }r\right]\\ a_{2}\left(t\right) & =\frac{1}{\left(1+\alpha^{2}\right)}\left[V_{c,1}\left(\alpha {\dot{\lambda }}_{1}+\frac{2\alpha }{\tau }\lambda_{1}\right)-V_{c,2}\left(\left(4\alpha^{2}+3\right){\dot{\lambda }}_{2}+\frac{2\alpha }{\tau }\lambda_{2}\right)+\frac{2\alpha }{\tau }r\right] \end{array} \end{array}$$

where the relation of line-of-sight angle (λ), line-of-sight rate $\left(\dot{\lambda }\right)$, closing velocity $\left(V_{c}\right)$ and time-to-go (τ) are given below:

(64) $$\begin{array}{cc} \dot{\lambda } & =\frac{\zeta +\dot{\zeta }\tau }{V_{c}\tau^{2}} \end{array}$$

(65) $$\begin{array}{cc} \lambda & =\frac{\zeta }{V_{c}\tau } \end{array}$$

Notice in Equation (63) that each agent is required to access the measurement information from both UAVs, to be able to compute the optimal solutions.

#### 3.2.2. Decentralized Solution with Information Deficiency Constraints

Based on Theorem 1 the decentralized solution of the leader problem can be obtained from Equation (62) as follows:

(66) $$\begin{array}{c} \begin{array}{cc} a_{1}\left(t\right) & =\frac{1}{\left(1+\alpha^{2}\right)}\left[-V_{c,1}\left(\left(3\alpha^{2}+4\right){\dot{\lambda }}_{1}+\frac{2}{\tau }\lambda_{1}\right)+V_{c,2|1}\left(\alpha {\dot{\lambda }}_{2|1}+\frac{2\alpha }{\tau }\lambda_{2|1}\right)-\frac{2}{\tau }r\right]\\ a_{2|1}\left(t\right) & =\frac{1}{\left(1+\alpha^{2}\right)}\left[V_{c,1}\left(\alpha {\dot{\lambda }}_{1}+\frac{2\alpha }{\tau }\lambda_{1}\right)-V_{c,2|1}\left(\left(4\alpha^{2}+3\right){\dot{\lambda }}_{2|1}+\frac{2\alpha }{\tau }\lambda_{2|1}\right)+\frac{2\alpha }{\tau }r\right] \end{array} \end{array}$$

and the follower problem is the well-known optimal rendezvous problem where the solution is given as:

(67) $$\begin{array}{c} \Delta a_{2}\left(t\right)=-\left[\frac{6}{\tau^{2}}\Delta \zeta_{2}+\frac{4}{\tau }\Delta {\dot{\zeta }}_{2}\right] \end{array}$$

which can be rewritten in terms of the line-of-sight angles as follows:

(68) $$\begin{array}{c} \Delta a_{2}\left(t\right)=-V_{c,2}\left[4{\dot{\lambda }}_{2}+2\frac{\lambda_{2}}{\tau_{2}}\right]+V_{c,2|1}\left[4{\dot{\lambda }}_{2|1}+2\frac{\lambda_{2|1}}{\tau_{2|1}}\right] \end{array}$$

and

(69) $$\begin{array}{c} \begin{array}{c} a_{2}\left(t\right)=a_{2|1}\left(t\right)+\Delta a_{2}\left(t\right) \end{array} \end{array}$$

Please note that the decentralized optimal strategy for agent_1_ can be computed using $\tau_{1}$ and $\lambda_{1}$ only, while the optimal strategy for agent_2_ requires all the measurement information from both UAVs.

#### 3.2.3. Potential Issues on Practical Implementation

The cooperative guidance laws proposed in this paper considers the matched time-horizon for both UAVs. However, they can vary for each agent because of the different launch conditions, uncertainties of the dynamical model, or external disturbances. To manage this issue, the cost function in Equation (45) can be extended for different time-horizons as follows:

$$J=E\left[\frac{b}{2}\left(\zeta_{1}^{2}\left(t_{f}\right)+\zeta_{2}^{2}\left(t_{f}\right)\right)+\frac{c}{2}{\left({\dot{\zeta }}_{1}\left(t_{f}\right)-\alpha {\dot{\zeta }}_{2}\left(t_{f}\right)-r\right)}^{2}+\frac{1}{2}\int_{0}^{t_{f,1}}\left(a_{1}^{2}\left(t\right)+a_{2}^{2}\left(t\right)\right)dt+\frac{1}{2}\int_{t_{f,1}}^{t_{f,2}}a_{2}^{2}\left(t\right)dt\right]$$

where the leader is assumed to intercept the target earlier than the follower does:

$$t_{f,1}\leq t_{f,2}$$

Before the time-to-go of the follower reach to the initial time-to-go of the leader, $V_{1}$ is assumed to be 0 that yields α=0. Therefore for $0\leq t\leq t_{f,2}-t_{f,1}$, the follower is guided with the proportional navigation (PN) guidance law for the centralized case and $u_{2|1}$ for the decentralized case, computed as follows:

$$u_{2|1}=-3V_{c,2|1}{\dot{\lambda }}_{2|1}$$

For $t\geq t_{f,2}-t_{f,1}$ the proposed guidance laws are used, where the leader predicts $V_{c,2|1}$, $\lambda_{2|1}$ and ${\dot{\lambda }}_{2|1}$ which depend on the time-to-go of the follower by using the dynamical propagation in Equation (12), whereas the follower retains the necessary information by communication networks or by direct measurements.

## 4. Numerical Experiments

In this section, the performance of the proposed guidance strategies are investigated on networked two-UAV systems. We consider the planar nonlinear kinematics for the UAVs as follows:

(70) $$\begin{array}{cc} {\dot{x}}_{i} & =V_{i}\cos\gamma_{i}\\ {\dot{y}}_{i} & =V_{i}\sin\gamma_{i}\\ V_{i}{\dot{\gamma }}_{i} & =a_{i}+w_{i} \end{array}$$

where the positions of the *i*-th UAV in the $X_{I}-Y_{I}$ frame from Figure 1 are denoted by $x_{i}$, $y_{i}$ in this section, and the other notations follow from Section 2. The process noise $w_{i}$ can be interpreted as the disturbance factor that includes the wind gust acting on the *i*-th UAV or target maneuver. Please note that it has been used and proven useful in a wide range of literature from classical optimal guidance problems [1,3,16,17,35,37].

In this example, two UAVs are launched from different locations with identical launch angle of 10 deg and guided to approach a stationary target with different approach angles separated by 30 deg. Please note that no explicit approach angle command for each UAV was given. For each UAV, the maximum available guidance command is assumed to be 3g, where *g* represents the gravitational acceleration. The initial states of the UAVs and the target are listed in Table 1.

Both the centralized and decentralized cooperation scenarios are considered here. In the centralized cooperation scenario, the deterministic UAV dynamics, i.e., $w_{i}=0$, in Equation (70) is considered for reference analysis. The two UAVs are assumed to share the perfect information on $\tau_{i}$, $\lambda_{i}$ and ${\dot{\lambda }}_{i}$ for i=1,2. In the decentralized cooperation scenario, the directional information constraint is considered, i.e., the first UAV’s sensor measurement is available to the second UAV, while the second’s measurement is not available to the first, and the stochastic dynamics is considered, where $w_{i}∼N\left(0,0.5^{2}\right)$ disturbs the dynamics and trajectories. Our main interest is to see whether the proposed decentralized cooperation scheme let the two UAVs approach the target with the required relative approach angle while making up the perturbed dynamics and trajectories caused by the random disturbance $w_{i}$’s.

### 4.1. Centralized Cooperation under Full Information Sharing

**Figure 3:**
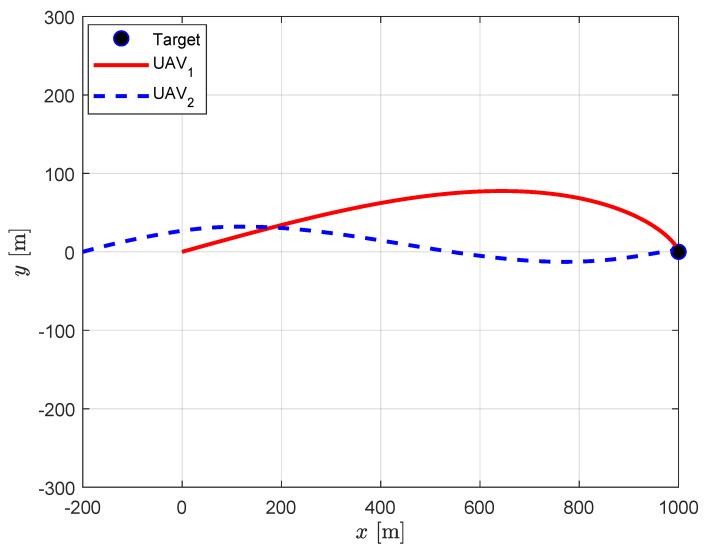
Centralized cooperation: UAV trajectories.

Figure 3 presents the trajectories obtained by the centralized guidance strategy. It is apparent that the guidance strategy enforces the required relative approach angle of 30 deg with a small error of 0.15 deg while both UAVs perfectly approaching to the target. Although both UAVs are launched to identical direction, UAV_1_ approaches the target from above while UAV_2_ approaches from below to satisfy the relative approach angle constraint. The impact time of each UAV is 10.12 s and 11.95 s, respectively. The detailed results are listed in Table 2.

**Figure 4:**
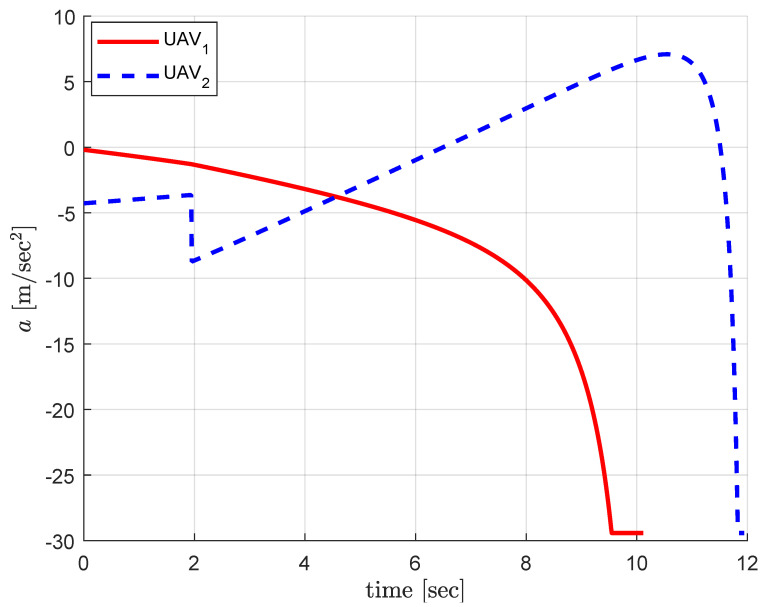
Centralized cooperation: guidance commands.

Figure 4 shows the acceleration commands during the engagement. The guidance command for UAV_1_ decreases to negative value which allows UAV_1_ to approach the target from above to satisfy the relative angle constraint. The guidance command for UAV_2_ increases from negative value to positive to approach the target from below to satisfy the relative approach angle constraint. Please note that UAV_2_ is guided with the PN guidance law for the first 1.95 s where the matched time-to-go for UAV_1_ does not exist in this engagement scenario. After that UAV_2_ cooperates with UAV_1_ for the matched time-horizon.

### 4.2. Decentralized Cooperation under Information Deficiency

**Figure 5:**
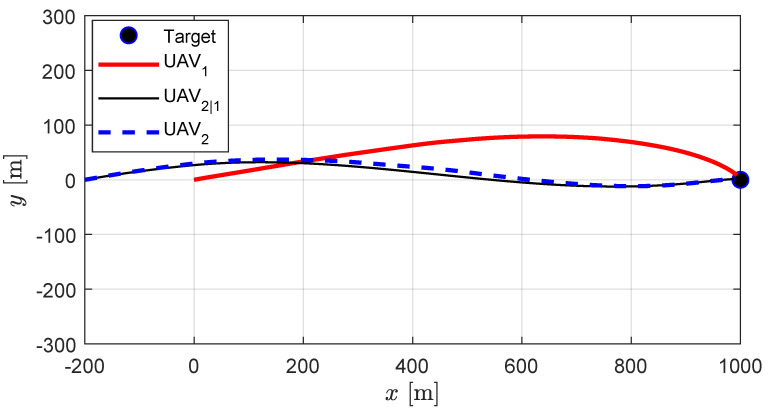
Decentralized cooperation: UAV trajectories. Thin black line represents UAV_1_’s best estimate on UAV_2_’s position, and the dotted blue line represents the actual trajectory of UAV_2_.

**Figure 6:**
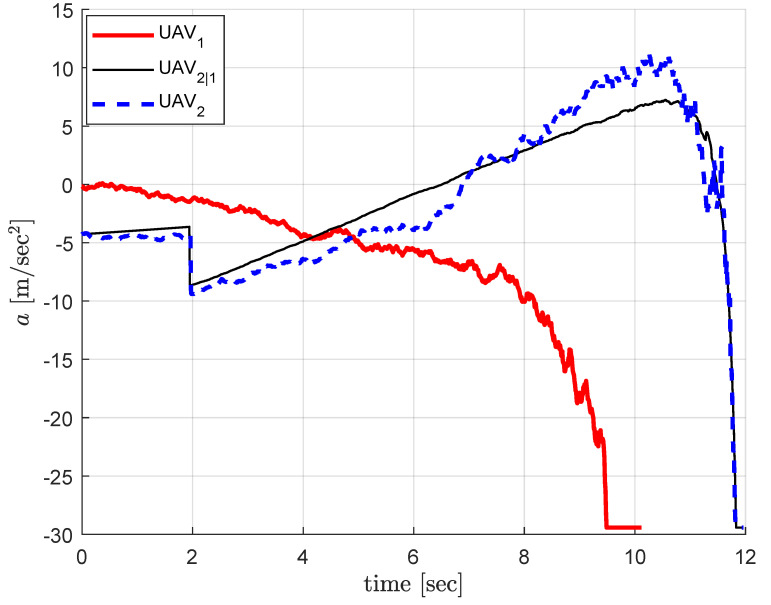
Decentralized cooperation: guidance commands. Thin black line (UAV_2|1_) represents UAV_1_’s best estimate on UAV_2_’s guidance command, and the dotted blue line (UAV_2_) represents the actual guidance command of UAV_2_ as the sum $a_{2}=a_{2|1}+\Delta a_{2}$.

Figure 5 and Figure 6 present the decentralized cooperation scenario. In this case, UAV_1_ estimates the state of UAV_2_ by the dynamic propagation and UAV_2_ issues a correction command in order to minimize the error caused by UAV_1_’s estimate. Figure 5 presents the trajectories obtained by the decentralized guidance strategy where the thin black line (UAV_2|1_) represents UAV_1_’s best estimate on UAV_2_’s position. It is evident that UAV_2_’s position converges to that of UAV_2|1_ which allows the relative approach angle to be maintained for a similar level with the centralized cooperation. The detailed results are listed in Table 3.

Figure 6 shows the acceleration commands during the engagement where the thin black line (UAV_2|1_) represents UAV_1_’s best estimate on UAV_2_’s guidance command. UAV_2_’s guidance command is maintained slightly less than that of UAV_2|1_ until UAV_2_’s correction command increases sufficiently, and become greater than the estimated value in order to making up the perturbed trajectories caused by the disturbance. It is obvious that the guidance command of UAV_2_ converges to that of UAV_2|1_ at the terminal time which indicates that the correction command converges to zero.

**Figure 7:**
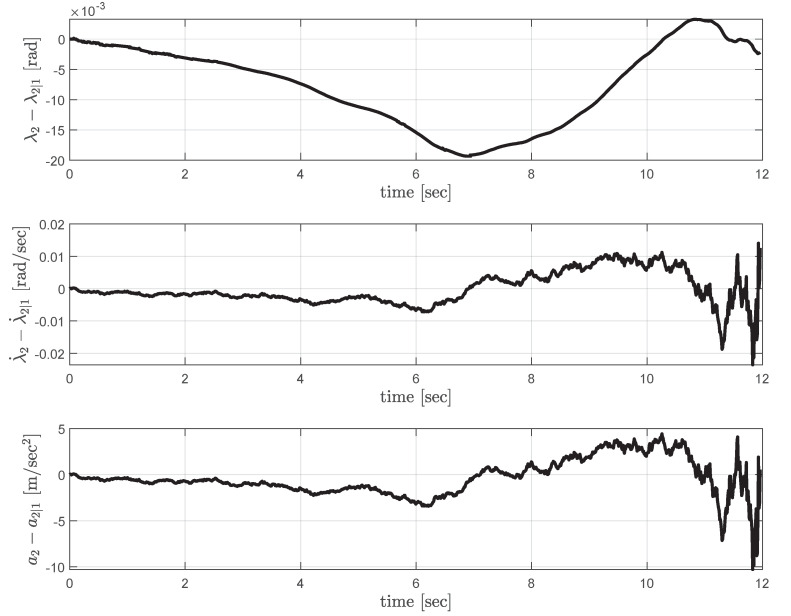
Decentralized cooperation: estimation error profiles (**top**, **middle**), and the correction command (**bottom**) computed by UAV_2_.

Figure 7 shows the error in UAV_1_’s estimate on UAV_2_’s line-of-sight angle and the line-of-sight rate. The last plot in Figure 7 shows the corresponding correction command computed by UAV_2_. The estimation errors increase under the influence of the disturbance, but they quickly converge to zero, which indicates the decentralized guidance strategy tries to follow the centralized guidance strategy. Please note that the fluctuating guidance commands observed when the time-to-go approaches to zero is usual as in most of the classical and practical guidance laws [1,3,16,17,35,37].

## 5. Conclusions

In this study, optimal cooperative guidance laws for two UAVs under sensor information deficiency constraints and the relative approach angle constraints are proposed. The general decentralized optimal control problem is formulated with the nested dynamics and information structure where the communication between the UAVs is directional. The optimal control problem is solved by adopting the state-separation principle which separates the decentralized optimal control problem into two centralized optimal control subproblems. The solution of the first subproblem considers the information deficiency which the leader is associated with according to the accessible information. The solution of the second subproblem considers additional effort for the follower that tries to minimize the prediction error of the leader. The optimal cooperative guidance solutions are derived in terms of the line-of-sight angles and the line-of-sight angle rates, so that it can be easily understood by the guidance community and can be easily implemented on conventional guidance computers.

Based on the proposed optimal cooperative guidance solution, the centralized and decentralized cooperative guidance laws are derived in closed form. Two UAVs cooperate to optimize a common objective function which couples their vertical velocity components at the terminal states. The performance of the proposed guidance strategies is investigated with nonlinear kinematics on both of centralized and decentralized cooperation setups. In the centralized cooperation setup, a deterministic scenario is considered, while a stochastic scenario is considered for decentralized cooperation setup. The simulation results show the guidance strategy enforces the required relative approach angle constraints and the decentralized guidance strategy converges to the centralized guidance strategy as the follower supports the prediction of the leader.

Future research directions may include extensions to general *n*-UAV cooperative guidance solutions or other cooperative mission objectives. Other realistic constraints such as collision avoidance, communication range, communication delay, and so on, should also be taken into account for practical implementation of the proposed approach.

## Figures and Tables

**Table 1:** Initial states for the numerical example.

Parameters	UAV_1_	UAV_2_	Target
$x(t_{0})$ [m]	0	−200	1000
$\gamma (t_{0})$ [deg]	10	10	-
$V(t_{0})$ [m/s]	100	100	0

**Table 2:** Results from the centralized cooperation with full information.

	UAV_1_	UAV_2_
Approach angle [deg]	−29.32	0.53
Impact time [s]	10.12	11.95

**Table 3:** Results from the decentralized cooperation with information constraints.

	UAV_1_	UAV_2_
Approach angle [deg]	−30.36	−0.47
Impact time [s]	10.12	11.96
